# Cycles of Abuse: The Mediating Role of Emotional Abuse on the Relationship Between Childhood Adversity and Depressive Symptoms

**DOI:** 10.1007/s40653-025-00722-0

**Published:** 2025-06-11

**Authors:** George Van Doorn, Dixie Statham, Jacob Dye, Robert Teese, Megan Jenkins

**Affiliations:** 1 Institute of Health and Wellbeing, Churchill Campus, Federation University Australia, Churchill, VIC 3842 Australia; 2 Health Innovation and Transformation Centre, Mt Helen Campus, Federation University Australia, Ballarat, VIC 3350 Australia; 3 Successful Health for At-Risk Populations (SHARP) Research Group, Mt Helen Campus, Federation University Australia, Ballarat, VIC 3350 Australia; 4 Institute of Health and Wellbeing, Mt Helen Campus, Federation University Australia, Ballarat, VIC 3350 Australia

**Keywords:** Depression, Emotional abuse, Adverse Childhood Experiences, Romantic relationships

## Abstract

Emotional abuse is associated with several deleterious outcomes including poor mental health, sexual assault, and partner homicide. Exposure to violence during childhood is an Adverse Childhood Experience (ACE) that has also been shown to increase the likelihood of experiencing mental health issues, including depression. Building on this understanding, this paper presents two studies that examined the relationship between these constructs, hypothesizing that individuals with ACEs would be more likely to experience both depressive symptoms and emotional abuse in intimate relationships during adulthood. Study One consisted of 345 Australian women and men aged 18 to 29 years, while Study Two comprised 700 women (18–82 years) from several countries. In both studies, participants completed online measures assessing ACEs, adult experiences of emotional abuse, and depression. Results from both studies showed that experiencing childhood adversity increases the risk of experiencing emotional abuse in intimate relationships and developing depressive symptoms in adulthood. Moreover, emotional abuse positively predicted depression, even after controlling for ACEs. The results provide further evidence for intergenerational cycles of abuse and their long-term mental health consequences.

## Introduction

Childhood adversity refers to potentially traumatic experiences in childhood such as physical and emotional abuse, household dysfunction, and neglect (Boullier & Blair, 2018; Mersky et al., 2017). According to Boullier and Blair (2018), Adverse Childhood Experiences (ACEs) are common with 64% of people experiencing at least one ACE. In North America, one-in-four children have experienced some form of maltreatment at the hands of a caregiver (Redd, 2017).

The impact of early adverse experiences extends well into adulthood, particularly in relation to mental health. Research has consistently shown that childhood adversity is a significant risk factor for depression, even when accounting for other contributing factors (Del Giudice, 2014). Chapman et al. (2004), for example, found a strong dose–response relationship between exposure to ACEs and lifetime depressive disorders. The findings of Chapman et al. (2004) were replicated in a study of Ethiopian adolescents (Tsehay et al., 2020). Further, systematic and umbrella reviews have demonstrated that childhood adversity is a key factor in lifelong mental health issues, including depression (Abate et al., 2025; Kalmakis & Chandler, 2015; Sahle et al., 2022). In combination, the evidence strongly suggests that experiencing adversity in childhood increases the likelihood of suffering from depression and other mental health issues later in life.

### Childhood Adversity and Emotional Abuse

Childhood adversity negatively impacts adult romantic relationships (Boullier & Blair, 2018; Redd, 2017). Experiencing ACEs increases the likelihood of individuals becoming both victims and perpetrators of Intimate Partner Violence (IPV) in adulthood. IPV includes physical, verbal, sexual, emotional, and economic abuse, as well as controlling behaviors like restricting access to friends (Cochran et al., 2011; Franklin & Kercher, 2012; Larsen, 2016; Mitchell, 2009; Renner & Whitney, 2012; World Health Organization, 2012; Yount et al., 2018). Exposure to IPV and physical punishment in childhood doubles the risk of experiencing or perpetrating IPV in adulthood (Ehrensaft et al., 2003; Franklin & Kercher, 2012).

This relationship between ACEs and IPV in adulthood has been shown to differ by gender. Women with ACEs face a higher risk of experiencing violence in their adult relationships, a pattern also seen in their daughters’ adolescent relationships (Adams et al., 2019; Alexander, 2009; Foran et al., 2014; Holmes et al., 2018; Rivas et al., 2020; Till-Tentschert, 2017). Notably, women who are sexually abused or exposed to abuse as children are more likely to experience physical, sexual, and emotional exploitation in adulthood (Iverson et al., 2011). Given the well-documented link between childhood abuse and women’s later revictimization, it is important to consider childhood adversity more broadly (Chu, 1992; Franklin & Kercher, 2012). Attachment theory provides a lens through which the link between ACEs and revictimization in adulthood can be explained from a developmental perspective.

Bowlby (1973, 1984) posits that the ‘blueprint’ for adult relationships is shaped by early childhood experiences. According to attachment theory, a child’s experience with their caregivers can impact their internal working models (i.e., cognitive and emotional representations of themselves and others; Bowlby, 1973). Consistent and responsive caregiving in early childhood promotes a secure attachment to the caregiver, characterized by trust and a sense of safety, and a schema for a secure attachment in adult relationships. Conversely, caregiving that is inconsistent, unreliable, rejecting, or chaotic can lead to the development of insecure attachments (Main & Solomon, 1986). Although early conceptualizations of adult attachment styles categorized individuals as falling into one of four distinct categories (i.e., secure, avoidant, anxious, disorganized), substantial evidence suggests a dimensional approach. Specifically, insecure adult attachment is best characterized as varying along two continuous dimensions of attachment anxiety and attachment avoidance (Fraley & Speaker, 2003; Fraley et al., 2015; Zhang et al., 2022).

Importantly, the experience of certain types of ACEs may be especially important in the formation of specific attachment styles. Meta-analytic findings suggest that emotional abuse is a strong predictor of insecure attachment (Baer & Martinez, 2006; Riggs & Kaminski, 2010), although it remains unclear whether emotional abuse differentially predicts anxious or avoidant attachment. Physical abuse has been linked to avoidant attachment, with survivors limiting emotional dependence to protect against further harm (Finzi et al., 2000, 2001; McCarthy & Maughan, 2010; Unger & De Luca, 2014). Neglect has been associated with both anxious and avoidant attachment, with some individuals developing a heightened need for reassurance (Benoit, 2004; Cassidy & Berlin, 1994), while others anticipate consistent unavailability and withdraw emotionally (Li & Wang, 2023; Venet et al., 2007). The extent to which ACEs shape insecure attachment underscores their influence on internal working models, ultimately shaping expectations of adult relationships and the degree of trust placed in others.

Attachment anxiety in adulthood may present as difficulty trusting others, a fear of abandonment or rejection, excessive approval seeking, a high sensitivity to a partner’s moods and actions, poor boundary setting, and distress at perceived rejection (Zhang et al., 2022). Attachment avoidance may also be associated with difficulty trusting others (Taunton et al., 2021) and fear of rejection, but may also present as a fear of dependence or intimacy, avoidance of self-disclosure, excessive self-reliance, and difficulty expressing emotion (Zhang et al., 2022). Insecurely attached adults may exhibit high levels of attachment anxiety, attachment avoidance, or both and have a higher likelihood of using defensive secondary strategies, including clingy and coercive behaviours, hypersensitivity to cues of rejection, refusal to acknowledge emotions, and jealousy in response to emotional discomfort or conflict in their relationships (Pollard & Cantos, 2021). Attachment anxiety and attachment avoidance are reliable predictors of IPV for both women and men (Babcock et al., 2000; Henderson et al., 2005; Pollard & Cantos, 2021). In summary, ACEs may be considered a risk factor for developing an insecure attachment style in adulthood which, in turn, is a risk factor for IPV. The link between ACEs and attachment style, therefore, provides a mechanism for understanding how a cycle of traumatic relationships may be perpetuated.

While IPV comprises multiple behaviors, a key component is non-violent emotional abuse – acts intended to isolate, denigrate, manipulate, terrorise, constrain, exploit, punish, reject, scapegoat, and control an intimate partner (Beck & Raghavan, 2010; Dutton & Goodman, 2005; Gou et al., 2019; Kelly & Johnson, 2008; Lehmann et al., 2012; Murphy & Hoover, 1999; Stark, 2012; Sweet, 2019). Research suggests that emotional abuse may be more prevalent than physical IPV, with at least equivalent psychological and social outcomes (Crossman et al., 2016; Dutton et al., 1999; Lischick, 1999; Stark & Hester, 2019).

The victims of non-violent emotional abuse are disproportionately female, but males also experience emotional abuse perpetrated by women (Perryman & Appleton, 2016; Robertson & Murachver, 2011; Tanha et al., 2010). Australian Bureau of Statistics (Australian Bureau of Statistics, 2020) data shows that one-in-four Australian women have experienced emotional abuse by a current or previous partner, with Queen et al. (2009) arguing that emotional abuse is “more relentless and terrorizing than physical abuse” (p. 237). However, emotional abuse is not only experienced by women. A recent systematic review of 19 papers from six countries showed that while emotional abuse was reported less frequently by men, a substantial number of men experienced emotional and psychological abuse from their female partners during the lockdown period of COVID-19 (Ononokpono & Uzobo, 2024). Concerningly, some studies have shown that controlling behavior and partner-instigated separation were significant predictors of intimate partner homicide (Campbell et al., 2003).

The forms of emotional abuse perpetrated by both women and men are similar, and include isolation, threats, sexual coercion, and gaslighting (Graham-Kevan et al., 2021; Ononokpono & Uzobo, 2024; Tanha et al., 2010). However, there appear to be behaviors that are more commonly directed towards men (e.g., false allegations to police, limiting contact with children, denigrating sexual performance; McHugh et al., 2013). Much like the sequelae of emotional abuse for women, the psychological outcomes for men appear severe and long-lasting (e.g., anxiety, depression, post-traumatic distress, suicidal ideation; Graham-Kevan et al., 2021).

## Emotional Abuse and Depression

Experiencing non-violent emotional abuse has negative psychological outcomes (Crossman et al., 2016; Dutton et al., 1999; Lischick, 1999; Stark & Hester, 2019). Men who are subjected to emotional abuse by their female partners are more likely to experience depression and suicidal thoughts than those not subjected to emotional abuse (Bates & Carthy, 2020; Gou et al., 2019; Simonelli & Ingram, 1998). With respect to the experiences of women, Campbell et al. (1997) showed that the bivariate correlation between non-physical abuse and depression was statistically significant (see Tahir et al., 2023 for a similar finding), while a multivariate analysis revealed that this relationship was not statistically significant. Dutton et al. (1999), however, showed that variables related to psychological abuse (i.e., dominance, isolation, and emotional and verbal abuse) were statistically significant when included in a multivariate model, and explained 27% of the variance in the severity of women’s depression. Of relevance, Li et al. (2024) found that psychological abuse was significantly associated with both depressive symptoms and risk of suicide in female college students.

A possible mechanism explaining the relationship between emotional abuse and depression is that individuals who experience emotional abuse suppress the expression of emotions (Zhou & Zhen, 2022). Suppressing emotions is thought to cause individuals to experience ambiguity with respect to their feelings, ruminate on the experience(s), fail to repair their mood, and view emotions negatively (Gross & John, 2003). Higher levels of depression are associated with these processes (Beevers & Meyer, 2004; Schafer et al., 2017).

## Childhood Adversity, Emotional Abuse, and Depression

Traumatic events in childhood have been associated with an increased risk of depression in adulthood, yet not all individuals exposed to traumatic events will experience depression. This discrepancy suggests that psychological constructs might mediate this relationship. Simpson et al. (2020), for example, established that self-disgust mediated the relationship between childhood trauma and psychosis (a dimension of which is depressive symptoms), while Wong et al. (2019) established that self-concept (i.e., the stability and coherence of a person’s sense of identity) mediated the relationship between childhood trauma and depression. However, the role of emotional abuse within intimate relationships remains underexplored.

## Current Study

Based on past research, we propose that emotional abuse in romantic relationships will mediate the relationship between childhood adversity and depressive symptoms. Given that emotional abuse predicts traumatic responses, the number of marriages and domestic partnerships has increased in recent years, the estimated annual cost of domestic violence in Australia is substantial, and witnessing violence can have serious life-long impacts on children (Access Economics, 2004; Australian Bureau of Statistics, 2018a, 2018b; Dutton et al., 1999), there is a pressing need to investigate the impact of emotional abuse. The key aim of this research was to determine whether childhood adversity increases risk of depression and whether this relationship is explained by experiences of emotional abuse in adult romantic relationships. To address this aim, we conducted two complementary studies. Study One assessed these relationships in an Australian cohort of young men and women, while Study Two assessed these relationships with an international cohort of women. Given previous findings, it was hypothesized that childhood adversity would be a direct, positive, and statistically significant predictor of depressive symptoms. Further, greater exposure to adverse events during childhood would predict higher exposure to emotional abuse in adult romantic relationships which, in turn, would be associated with increased depressive symptoms. The research design, employing cross-sectional surveys and mediation analyses, allowed us to efficiently collect data from large, diverse samples and examine both direct and indirect associations between childhood adversity and depressive symptoms.

## Method

### Participants

#### Study One

**Fig. 1:**
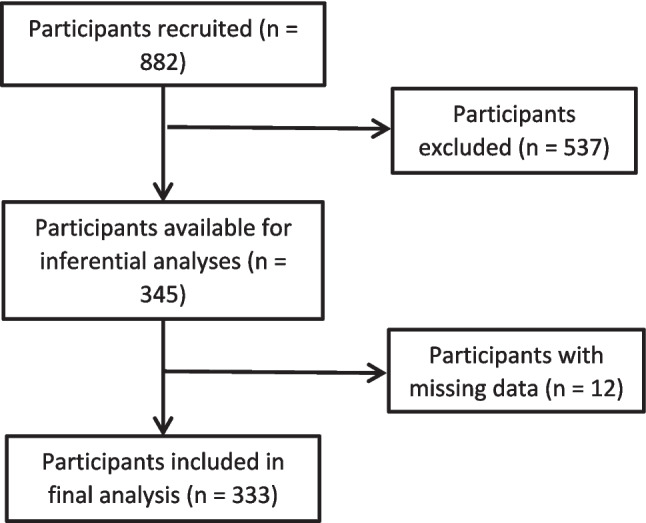
Flowchart depicting the participant selection process

A total of 882 Australian adults accessed the survey through recruitment website Prolific. Following the application of exclusion criteria, 537 were excluded from the analysis. The reasons for exclusion included not answering any questions (*n* = 6), only completing demographic questions (*n* = 75), failing to provide information about their current relationship status (*n* = 10), or not being in a romantic relationship which was necessary to answer questions about a current romantic partner’s use of emotional abuse (*n* = 446). The final sample consisted of 345 respondents (39.1% of the sample; see Fig. 1) who ranged in age from 18 to 29 years (*M* = 23.38, *SD* = 3.46). In terms of gender identity, 22.9% (*n* = 79) identified as men, 74.5% (*n* = 257) identified as women, and 2.6% (*n* = 9) identified as non-binary or gender-fluid.

#### Study Two

**Fig. 2:**
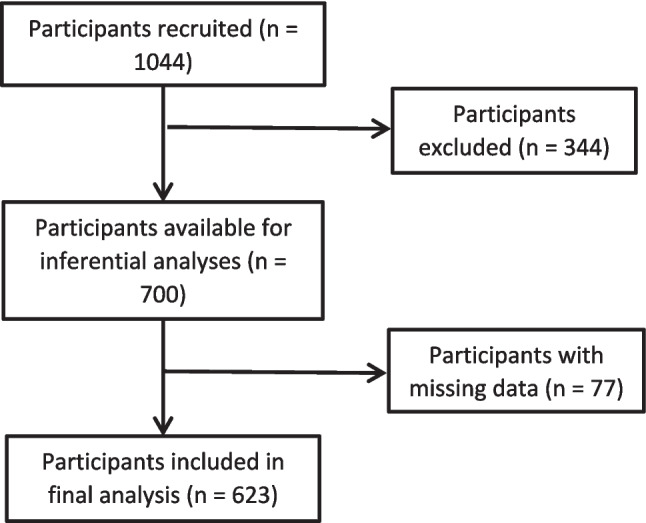
Flowchart depicting the participant selection process

A total of 1044 people from various countries accessed the survey via Prolific. The majority were from the United Kingdom (including Scotland, Wales, and England; 85.4%), with smaller proportions from New Zealand, Canada, Finland, Iceland, Norway, and Sweden. After applying exclusion criteria, 344 people were excluded from the analysis. Reasons for exclusions included identifying as a man (*n* = 6), identifying as non-binary or gender-fluid (*n* = 7), failing to answer anything other than demographics questions (*n* = 26), not being in a romantic relationship (*n* = 294), or failing to provide information regarding their relationship status (*n* = 11). The final sample comprised 700 women (67.1% of respondents) with an age range of 18 to 82 years (*M* = 36.16, *SD* = 11.87; see Fig. 2).

## Materials

### Adverse Childhood Experiences

The Childhood Experiences Survey (CES; Mersky et al., 2017) consists of 17 items; 10 measure two domains of ACEs: childhood maltreatment (neglect and physical, emotional, and sexual abuse) and household dysfunction (alcohol/drug problems, mental illness, domestic violence, incarceration, divorce/separation). The remaining seven items measure other childhood adversities: family financial problems, food insecurity, homelessness, parental absence, peer victimisation, being the victim of violent crime. Responses are obtained using a five-point Likert scale for seven items (0 = *never*, 4 = *very often/always*), a three-point Likert-type scale for three items (0 = *never to* 2 = *more than once*), or a binary scale for seven items (1 = *yes*, and 0 = *no*). Response scales were altered such that the response options and anchors, and thus the weightings, of each question were similar (e.g., 4 = *Yes*, 0 = *No*) and a total score was calculated by summing all item scores. Higher total scores reflect greater range and frequency of ACEs. The CES has good internal reliability (Cronbach’s α = 0.81; Mersky et al., 2017) and, in the current study, internal consistency was found to be acceptable in Study One (α = 0.78) and good in Study Two (α = 0.80).

### Emotional Abuse

The Multidimensional Measure of Emotional Abuse (MMEA; Murphy & Hoover, 1999) was used to assess how frequently a participant’s romantic partner had engaged in emotional abuse in the past 6 months. An example item is “*Your partner: Secretly searched through the other person’s belongings?*”. The 28-items are answered on seven-point Likert-type scales ranging from 0 (n*ever*) to 6 (*11–20 times*). A total score was calculated by summing all item scores. Higher total scores indicate more frequent exposure to emotional abuse. The MMEA has been shown to have excellent internal reliability (Cronbach’s α = 0.92; Godfrey et al., 2021). The MMEA’s internal consistency was excellent in both Study One (α = 0.92) and Study Two (α = 0.94).

### Depressive Symptoms

The Patient Health Questionnaire – 9 (PHQ-9; Kroenke et al., 2001) assessed participants’ levels of depressive symptoms over the past two weeks. Participants responded to the nine items using a four-point Likert-type scale ranging from 0 (*Not at all*) to 3 (*Nearly every day for two weeks*), and a total score was obtained by summing the item scores. Higher total scores indicate a greater likelihood that the person has depressive features. According to Kroenke et al. (2001), the optimal PHQ-9 cut-off value to screen for major depressive disorder is ≥ 10 (sensitivity 88%; specificity 88%). The PHQ-9 has good internal consistency (Cronbach’s α = 0.86–0.89; Kroenke et al., 2001), and in both of our studies the reliability was excellent (Study One: α = 0.90; Study Two: α = 0.91).

## Design and Statistical Analysis

A cross-sectional design was used in both studies, and two mediation analyses were conducted using SPSS 28.0.1.0 and Version 3.5 of the PROCESS Macro (Hayes, 2018). The predictor variable in both studies was ACEs, while the mediating variable was emotional abuse. The outcome variable in both studies was depressive symptoms. No covariates were included in the analyses. We subjected our analyses to Bonferroni corrections and, as there were two tests (i.e., Australian emerging adults, international women), we applied the stringent critical *p*-value of 0.05/2 = 0.025.

An a priori power analysis conducted using Monte Carlo Power Analysis for Indirect Effects (Schoemann et al., 2017) with 2 predictors, *p* = 0.05, power = 0.80, and a moderate effect size = 0.25, determined that a sample size of 224 people was needed to achieve sufficient power. As the final sample of each study comprised more individuals than this, the assumption of sample size was met.

## Procedure

Participants were recruited using Prolific. Weblinks and Plain Language Statements were posted on Prolific and inclusion/exclusion criteria were specified by the researchers. In Study One, these criteria were: 18–29 years of age, could read English, and were Australian citizens/permanent residents. In Study Two, these criteria were: the individual identified as a woman and could read English. Participants who met the eligibility criteria and chose to participate were directed from Prolific to the survey in Qualtrics. After reading an explanation of the study and agreeing to participate, the survey began with demographic questions including age, gender, marital status, education, and income. Participants then responded to the questions assessing ACEs, depressive symptoms, and experiences of emotional abuse in the current, romantic relationship. Upon completion, participants were redirected back to Prolific to receive payment. Each survey took approximately 15 min to complete, and all participants received either ~ AUS$9 (Study One) or ~ AUS$13 (Study Two).

## Results

### Assumption Testing

#### Study One

Four multivariate outliers were identified and removed. All other assumptions were met. Missing values were present in the dataset due to non-response, with the proportion of missing values ranging from 0.6% to 1.2%. In both Study One and Study Two, multiple imputation was initially performed to address missing data, and the inferential analysis was conducted both with and without imputed values. As the results remained substantively unchanged, the original dataset was retained, and the mediated regression analyses were conducted using listwise deletion. This approach was preferred due to the limitations of the PROCESS macro in handling imputed data and because, given the nature of the data being collected, one could interpret non-response as an indication of the participant revoking their consent.

#### Study Two

The residuals were slightly positively skewed. As an assumption of regression is that the residuals are normally distributed, data from participants whose residuals were greater than 3 SDs from zero were removed. This resulted in the data from six participants being removed. Eight multivariate outliers were also identified, and data from these participants were removed. No other assumptions were violated. Following the removal of these individuals, there were missing values in the dataset due to non-response, with a higher proportion than in Study One (1.4 – 8.6%).

## Descriptive Statistics

### Study One

Descriptive statistics and bivariate correlations are presented in Table 1. The bivariate correlations suggest that ACEs were positively associated with both an individual’s experience of emotional abuse in a current relationship and depressive symptoms.

**Table 1:** Correlation matrix between adverse childhood experiences, emotional abuse, and depressive symptoms

	M	SD	ACEs	MMEA	PHQ
ACEs	15.50	10.63	-	.23***	.37***
MMEA	14.73	16.57			.23***
PHQ	10.20	6.56			-

ACEs = Adverse Childhood Experiences, MMEA = Multidimensional Measure of Emotional Abuse, PHQ = Patient Health Questionnaire, ***p < .001

Using a cut point of ≥ 10 for the PHQ-9 (Kroenke et al., 2001), 44.4% of the sample screened positive for probable depression.

### Study Two

Descriptive statistics and bivariate correlations are presented in Table 2. The bivariate correlations suggest that ACEs were positively associated with both an individual’s experience of emotional abuse in a romantic relationship and depressive symptoms.

**Table 2:** Correlation matrix of adverse childhood experiences, emotional abuse, and depressive symptoms

	M	SD	ACEs	MMEA	PHQ
ACEs	13.97	10.98	-	.10**	.34***
MMEA	13.01	18.07		-	.32***
PHQ	7.85	6.37			-

ACEs = Adverse Childhood Experiences, MMEA = Multidimensional Measure of Emotional Abuse, PHQ = Patient Health Questionnaire, **p < .01, ***p < .001

Using a cut point of ≥ 10 for the PHQ-9 (Kroenke et al., 2001), 28.7% of the sample screened positive for probable depression.

## Mediation Analyses

### Study One

**Fig. 3:**
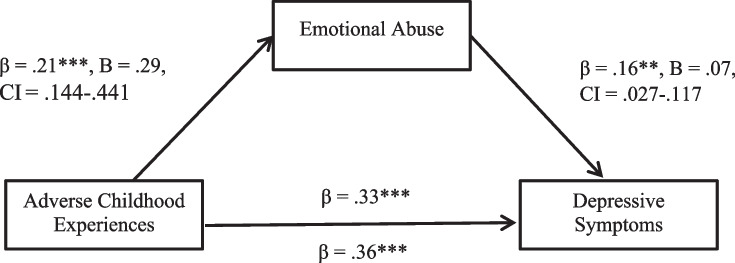
The statistical model demonstrating how emotional abuse mediates the effect of ACEs on depressive symptoms. Note: **p < .01, ***p < .001

The model was statistically significant overall, *F*(2, 330) = 30.03, *p* < 0.001, *R*^2^ = 0.15. The mediation analysis revealed a statistically significant indirect association between ACEs and depressive symptoms, mediated by emotional abuse (β = 0.03, CI_bootstrapped_ = 0.008 to 0.068). The remaining direct association between ACEs and depressive symptoms was statistically significant (β = 0.33, B = 20, CI_bootstrapped[unstandardised]_ = 0.139 to 0.266, *p* < 0.001). Thus, the association between ACEs and depressive symptoms (β = 0.36, B = 22, CI_bootstrapped[unstandardised]_ = 0.161 to 0.286, *p* < 0.001) can be partly explained by an indirect association via emotional abuse. There is some evidence to suggest that, in young Australian adults, increased childhood adversity is associated with greater experiences of emotional abuse in a current, romantic relationship which, in turn, is associated with more symptoms of depression (Fig. 3).

### Study Two

**Fig. 4:**
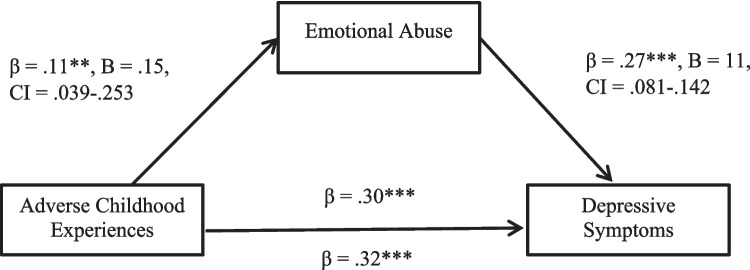
The statistical model demonstrating how emotional abuse mediates the effect of the adverse childhood experiences on depressive symptoms. Note: **p < .01, ***p < .001

The model was statistically significant overall, *F*(2, 620) = 65.29, *p* < 0.001, *R*^2^ = 0.17. The mediation analysis revealed a statistically significant indirect association between ACEs and depressive symptoms when mediated by emotional abuse (β = 0.03, B = 0.17, CI_bootstrapped_ = 0.007 to 0.053). The remaining direct association between ACEs and depressive symptoms was statistically significant (β = 0.30, B = 0.17, CI_bootstrapped[unstandardised]_ = 0.129 to 0.212, *p* < 0.001). Thus, the association between the ACEs and depressive symptoms (β = 0.32, B = 0.19, CI_bootstrapped[unstandardised]_ = 0.143 to 0.229, *p* < 0.001) is partly explained by an indirect association via emotional abuse. Given the results, there is some evidence to suggest that ACEs are associated with increased experiences of emotional abuse in a current, romantic relationship which, in turn, is positively associated with depressive symptoms (Fig. 4).

## Discussion

The present study investigated whether childhood adversity increases the risk of depression, and whether this relationship is explained by experiences of partner emotional abuse. Building on previous research, it was hypothesized that experiencing adverse events in childhood would be a direct, and positive, predictor of depressive symptoms. This hypothesis was supported in young Australian men, women, and non-binary individuals, and in an international cohort of women. The hypothesis that those people who experienced more childhood adversity would experience more emotional abuse in a current, romantic relationship which would, in turn, be positively associated with depressive symptoms was also supported.

In both young Australian adults and an international cohort of women, ACEs were a statistically significant predictor of depressive symptoms, which is consistent with previous research (Abate et al., 2025; Chapman et al., 2004; Kalmakis & Chandler, 2015; Sahle et al., 2022). We found that self-reported childhood adversity uniquely explained variance in depressive symptoms in both cohorts, even after controlling for emotional abuse. Thus, experiencing maltreatment and household dysfunction, amongst other adversities, when growing up appears related to adult depressive symptoms. Early recognition and prevention of ACEs, and early treatment for those affected by ACEs, may be crucial in preventing depressive disorders.

Consistent with previous findings, the experience of childhood adversity increased the likelihood that individuals would be emotionally abused in adult romantic relationships (Cochran et al., 2011; Franklin & Kercher, 2012; Iverson et al., 2011; Renner & Whitney, 2012; Yount et al., 2018). Although not the focus of this study, past work has suggested that those who experience childhood adversity may come to expect that their emotional needs will not be met adequately in adult relationships, or that their characteristics (e.g., hostility, anxious attachment style) may negatively influence the relationship (Pilkington et al., 2021; Sun et al., 2021). Interestingly, ACEs explained more of the variance in the emotional abuse experienced by young Australian adults than it did in a cohort of international women. Although speculative, one possible explanation for this finding is the age differences of the samples. The international cohort was, on average, older than the Australian cohort, and thus the influence of ACEs was less proximal. It may be that observing or experiencing emotional abuse in childhood socialises young people to expect emotional abuse in romantic relationships in early adulthood (see Richards et al., 2017, for a similar argument with respect to violence), but the traumatic experiences in women’s early romantic relationships may encourage them to choose more trustworthy and sincere romantic partners later in life (Gobin, 2012). Another possibility is that as women age, and thus spend more time in emotionally abusive relationships, they become desensitised to emotional abuse (see Mullin & Linz, 1995). Thus, although the level of emotional abuse remains relatively unchanged across time (see Mezey et al., 2002), women’s perceptions may change.

The mediation analysis showed that, in both samples, emotional abuse positively predicted depression, even after controlling for ACEs. Our findings are consistent with, and build on, evidence that experiencing non-violent emotional abuse has negative psychological outcomes (Crossman et al., 2016; Dutton et al., 1999; Lischick, 1999; Stark & Hester, 2019). Men who are subjected to emotional abuse by their female partners, for example, are more likely to experience depression (Gou, et al., 2019; Simonelli & Ingram, 1998), while Dutton et al. (1999) showed that variables related to psychological abuse (e.g., isolation, emotional and verbal abuse) explained variance in the severity of women’s depression. Our findings suggest that experiencing emotional abuse in romantic relationships is harmful for all adults’ mental health, even after controlling for childhood adversity.

A critical consideration is how these findings can be generalized across populations and contexts. Our study utilized samples drawn from young Australian adults and an international cohort of women, and consistent patterns across these groups suggest that the identified relationships between childhood adversity, emotional abuse, and depressive symptoms may extend beyond our study samples. Specifically, the fact that similar mediation effects were found across culturally and demographically distinct groups implies that the impact of early adverse experiences on later revictimization and mental health outcomes may be present in other groups.

## Limitations and Future Directions

While this study provides novel insights into the role emotional abuse plays in mediating the relationship between ACEs and depressive symptoms, several methodological limitations should be considered. One limitation is the study’s cross-sectional design, which inhibits the ability to establish causality. Although our study has demonstrated that the relationship between ACEs and depressive symptoms is mediated by emotional abuse, and there is existing evidence that suggests ACEs lead to adult depressive symptoms, we caution against attributing causality because alternative explanations are possible (e.g., the expression of depressive symptoms may trigger greater emotional abuse). Although previous work suggests the direction of the relationships are supported, future studies could employ longitudinal designs to provide stronger evidence regarding causal pathways.

A second limitation is the relatively small proportion of variance explained by emotional abuse in the association between ACEs and depressive symptoms. As such, it would be useful to identify other mediating variables that might further explain the relationship between ACEs and depressive symptoms. That said, explaining only a small amount of the association is common in psychological research, particularly when studying complex, multifaceted constructs such as ACEs and depressive symptoms. Future research could explore additional mediators (e.g., emotion regulation difficulties, maladaptive coping strategies, social support) that may further explain this relationship.

Future research could also establish whether the relationship between individual ACEs (e.g., sexual abuse) and depressive symptoms are mediated by emotional abuse. As ACEs are interrelated (Anda et al., 1999; Felitti et al., 1998), we thought it important to consider them as a set of experiences that potentially affect depressive symptoms. That said, specific types of childhood adversity may have distinct psychological consequences, and it is worthwhile investigating whether emotional abuse serves as a mediator for some types of ACEs but not others.

## Implications and Conclusions

This work is, to the best of our knowledge, the first to assess the role that emotional abuse might play in explaining the relationship between childhood adversity and depressive symptoms. The two studies demonstrate that those individuals who experience more childhood adversity, regardless of gender, are more likely to be in romantic relationships with emotionally abusive partners which, in turn, is positively associated with experiencing depressive symptoms. These results have important implications for parental education programs and professional therapeutic practice. Mental health advocates and therapists may benefit from an increased awareness of the relationship between ACEs and behaviors within romantic relationships (e.g., emotional abuse), and their contribution to depressive symptoms. Further, when people engage with mental health practitioners, screening for these factors may assist therapists in developing the goals of any intervention. Raising awareness may encourage parents engaging in potentially adverse behaviors to seek professional help. We conclude that people who experience more adversity in childhood are at greater risk of experiencing more severe depressive symptoms, and that this relationship is explained (at least in part) by the emotional abuse experienced in a current, romantic relationship.

## Data Availability

Data are available upon reasonable request to the corresponding author.
